# A synchronous spectrofluorometric technique for simultaneous detection of alfuzosin and tadalafil: applied to tablets and spiked biological samples

**DOI:** 10.1098/rsos.220330

**Published:** 2022-07-13

**Authors:** Heba Samir Elama, Shereen M. shalan, Yasser El-Shabrawy, Manal I. Eid, Abdallah M. Zeid

**Affiliations:** Pharmaceutical Analytical Chemistry Department, Faculty of Pharmacy, Mansoura University, Mansoura 35516, Egypt

**Keywords:** tadalafil, alfuzosin, lower urinary tract symptoms, spectrofluorometry, biological samples

## Abstract

A facile, accurate, eco-friendly and sensitive spectrofluorometric method was evolved to assay alfuzosin hydrochloride (AFH) and tadalafil (TDF) in different matrices. Such a co-administered combination is clinically used for the treatment of lower urinary tract symptoms. Both compounds are characterized by their native fluorescence spectra upon excitation at specific wavelengths. Their characteristic fluorescence spectra were used for sensitive assay of the studied analytes in tablets and human biological samples. The assay principle is based on first-order synchronous spectrofluorometric scan using Δ*λ* = 60 nm in which AFH peaks were recorded at 366 nm. Meanwhile, TDF measurements were recorded at 293 nm in the same scans without overlap with AFH spectra. Recent analytical chemistry trends were implemented to lessen occupational and environmental perils, using ethanol as a diluting solvent for method optimization and application. Linearity ranges were 5.0–90.0 and 10.0–100.0 ng ml^−1^ for AFH and TDF, respectively in their raw materials with average % recoveries of 100.44% and 99.73% in raw materials, 100.15% and 100.20% in spiked plasma, and 97.14% and 99.99% in spiked urine. The proposed method was successfully applied to Prostetrol and Starkoprex commercial tablets with no interference with common tablet additives.

## 1. Introduction

Elderly men and women are recently suffering from lower urinary tract signs with reported elevated rates. The causes behind these symptoms are different and include common elderly male progressive disease benign prostatic hyperplasia (BPH). Additionally, BPH may be of life-threatening consequences [1,2].

Because of greater uroselectivity and minimal haemodynamic adverse effects, *α*1-blockers (alfuzosin hydrochloride (AFH), tamsulosin and silodosin) are the mainstay in symptomatic therapy of BPH. Though AFH is not completely *α*1A selective, it shows greater uroselectivity, whereas tamsulosin and silodosin have higher *α*1A selectivity [3,4].

**Figure 1.**
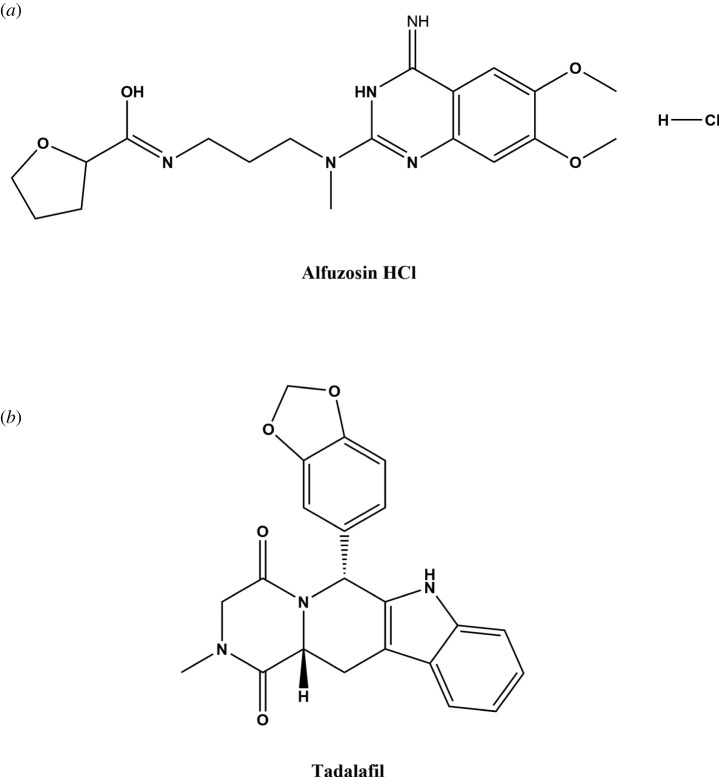
Chemical structures of alfuzosin hydrochloride and tadalafil.

AFH, figure 1*a*, is an *α*1-blocker whose chemical name is N-(3-((4-amino-6,7-dimethoxy-2-quinazolinyl)methylamino)propyl)tetrahydro-2-furancarboxamide; hydrochloride. It has a muscle-relaxing activity on smooth muscles that could be used for treatment of BPH [5]. Clinically, AFH binds selectively to *α*1 receptor alpha-1 in the bladder's smooth muscles. Hence, it instigates the relaxation of smooth muscles at the bladder and prostatic urethra, and then the symptoms of BPH and urine flow are relieved [6].

Tadalafil (TDF), figure 1*b*, is a selective PDE5 inhibitor whose chemical name is (2R,8R)-2-(2H-1,3-benzodioxol-5-yl)-6-methyl-3,6,17-triazatetracyclo[8.7.0.0^3,8^.0^11,16^]heptadeca-1(10),11,13,15-tetraene-4,7-dione [7]. It is a vasodilating agent used for the management of erectile dysfunction (ED) in elderly men. TDF is responsible for the accumulation of cGMP by selectively inhibiting PDE5 which in turn causes depletion of cGMP. Then, in the penial corpus cavernosa and spongiosum, cGMP gets accumulated leading to blood engorgement due to smooth muscle vasodilatation. As a result, penile erection is prolongated [8].

Phosphodiesterase type 5 inhibitors (PDE5 inhibitors) are commonly prescribed for the treatment of male erectile dysfunction [9]. Additionally, PDE5 inhibitors such as TDF have also shown improvement in the symptoms of BPH [10]. The combinations of α-blockers and PDE5 inhibitors have been assessed in different studies for treatment of lower urinary tract symptoms (LUTS) and ED. Such combination therapy with α-blockers/PDE5 inhibitors was found to be more effective than monotherapy with PDE5 inhibitors as specified by the International Index of Erectile Function scores, International Prostate Symptom Score and maximum flow rate [11–13].

In patients on AFH therapeutic system for BPH, its co-administration with TDF showed the greatest improvement of both BPH and erectile dysfunction symptoms compared with single-dose therapy with either an alpha-1 receptor antagonist or a PDE inhibitor [12,14].

The current work aims to develop an environmentally friendly and sensitive method for simultaneous estimation of AFH and TDF in their laboratory prepared mixtures and further application to detect both studied analytes in spiked human plasma and urine that were chosen due to oral absorption of AFH and TDF and their urine excretion. The method application in spiked biological samples aimed to test the method specificity in presence of plasma and urine components. The proposed method showed no interference with plasma and urine components confirmed by the satisfying mean recoveries in spiked plasma and urine samples.

By reviewing the previous literature, several methods were reported for the determination of AFH and TDF. For determination of AFH, HPLC [15–19], spectrophotometric [20–25], spectrofluorometric [25–28] and voltammetric [29–31] procedures were revealed. For TDF determination, different HPLC [32–34], spectrofluorometric [35–38], spectrophotometric [35,39–42] and voltammetric [43–46] methods were reported for its analytical quantitation.

To our knowledge, no analytical methods have been reported for simultaneous assay of the mixture under study and one HPLC method was reported for analysis of AFH, tamsulosin and vardenafil, which is a similar drug to TDF [47]. A spectrofluorometric method is reported for simultaneous quantitation of TDF and avanafil (PDE-5 inhibitor) in their combined tablet and spiked human plasma samples [48]. The main advantage of our method compared with the latter, regardless of sensitivity, is the greenness and simplicity, as we used ethanol compared with methanol and Britton-Robinson buffer by the reported one.

Green chemistry aims to meet environmental and economic goals simultaneously, and it has 12 principles [49]. The proposed method aimed to meet green chemistry goals, and method greenness was assessed by different assessment tools including analytical Eco-Scale [50], green analytical procedure index [51], and analytical greenness metric approach [52]. The type of applied technique determines the suitable assessment tools.

## 2. Experimental

### 2.1. Instrument

All measurements have been performed by an Agilent^®^ Cary Eclipse spectrofluorometer to which a xenon flash lamp is equipped. A smoothing factor of 20, a 5 nm slit width, and an applied voltage of 800 V were used during all experimental trials. Relative fluorescence intensities (RFI) of the estimated analytes were measured by first-order synchronous spectrofluorometric scan using Δ*λ* = 60 nm. AFH's synchronous peaks were constructed at 366 nm, and TDF measurements were recorded at 293 nm after converting synchronous scans into their first derivative.

### 2.2. Reagents and materials

Researchers were supplied with raw materials of AFH and TDF from RAMEDA, Cairo, Egypt with purities of 99.95% and 99.79%, respectively.

Commercial studied tablets were bought from local pharmacies and included Prostetrol tablets with a content of 10 mg AFH per tablet and Sterkoprex tablets containing 5 mg TDF per tablet.

Analytical grades of tween 80, sodium dodecyl sulfate, carboxymethyl cellulose and HPLC grade acetonitrile, isopropanol, ethanol, n-propanol and methanol were all obtained from Sigma-Aldrich, Cairo, Egypt.

Hospitals of Mansoura University, Dakahleya, Egypt supplied researchers with frozen plasma of a 26-year-old healthy volunteer and kept it at (−5°C) till used. While a fresh sample of urine was donated by a drug-free volunteer 29 years old.

### 2.3. Standard solutions

Stock standard solutions of AFH and TDF (100 µg ml^−1^ each) were prepared by accurately weighing 10 mg of the corresponding raw material, quantitatively transferring them to a 100 ml volumetric flask, and using ethanol as a solvent to complete to the mark. By appropriate dilution of prepared stock solutions using ethanol as a diluting solvent, studied concentration ranges were obtained.

### 2.4. Analytical procedures

#### 2.4.1. Calibration graphs

Two separate sets of 10 ml volumetric flasks were set (a set for AFH and the other for TDF), into which exact measured aliquots of corresponding stock solutions were separately transferred to cover the final diluted concentrations (table 1). All volumetric flasks were then completed to a final 10.0 ml volume with ethanol and mixed well. Afterward, against ethanol as blank, synchronous scans of AFH and TDF were measured using Δ*λ* of 60 nm with a filter size of 20 and an interval of 5. First-order derivative was applied. AFH measurements were conducted at 366 nm while TDF measurements were recorded at 293 nm. Calibration graphs were developed by plotting ΔF values against the corresponding drug's final concentration in ng ml^−1^. Otherwise, the matching regression equations have been set.

**Table 1.** Obtained data for the proposed spectrofluorometric method. *S*_b_, SD of the slope; *S*_a_, s.d. of the intercept; *S*_y/x_, s.d. of the residuals.

parameter	AFH	TDF
concentration range (ng ml^−1^)	5–90	10–100
LOD (ng ml^−1^)	0.72	1.52
LOQ (ng ml^−1^)	2.18	4.61
correlation coefficient (*r*)	0.9999	0.9999
intercept	1.26	0.08
slope	−0.5	−0.20
*S*_y/x_	0.17	0.12
*S*_a_	0.11	0.09
*S*_b_	0.002	0.001
% error	0.47	0.59
% RSD	1.23	1.32
mean found (%)	100.44	99.73
±standard deviation (s.d.)	1.24	1.32

#### 2.4.2. Applications

##### 2.4.2.1. Laboratory-prepared mixtures analysis

Taking into account the final dilution concentration ranges of the studied drugs shown in table 1, calculated aliquots of AFH and TDF stock solutions were carefully placed into a series of 10 ml volumetric flasks, diluted to the mark with the suitable solvent and mixed well. Then measurement procedure under §2.4.1 was followed. Table 2 summarizes the obtained % recoveries of the laboratory-tested synthetic mixtures. Per cent recoveries were calculated either using the constructed calibration graphs or the corresponding regression equations.

**Table 2.** Method application to raw materials and synthetic mixtures of AFH and TDF compared with their comparison methods.

	raw materials					
	proposed method				comparison methods [25,36]	
	amount taken (ng ml^−1^)	% found^a^	amount taken (ng ml^−1^)	% found^a^	% found^a^	
compound	AFH		TDF		AFH	TDF
	5	102.16	10	98.13	101.71	98.27
	10	100.95	20	100.67	99.44	100.24
	20	101.28	40	98.82	99.50	101.42
	40	98.23	60	101.36	100.24	99.25
	60	100.42	100	99.66		
	80	99.94	—	—		
	90	100.12	—	—		
Student-*t*					0.29 (2.26)	0.07 (2.45)
*F*					1.37 (8.94)	1.05 (5.95)
synthetic mixtures						
	amount taken (ng ml^−1^)		% found^a^			
compound	AFH	TDF	AFH	TDF		
	10	20	100.23	98.08		
	20	40	99.75	98.60		
	60	60	99.29	99.15		
mean ± s.d.			99.76 ± 0.47	98.60 ± 0.54		
s.e.			0.27	0.31		

^a^Each result is an average value of three separate determinations.

##### 2.4.2.2. Analysis of TDF and AFH in their commercial tablets

This application aimed to practically examine the average tablet content following ICH guidelines [53]. First, 10 tablets of Prostetrol/Starkoprex were weighed (each identical set was weighed separately) to calculate the average tablet weight. Then, each series was ground finely, well mixed, and the average tablet weight was quantitatively transferred to a 100 ml volumetric flask. Each flask content was then completed roughly to 50 ml with ethanol. After this, the two flasks corresponding to Prostetrol^®^ and Starkoprex^®^ were sonicated for half an hour to make sure that the extraction process has completed, and finally flasks were completed to the mark with ethanol. To remove the suspended tablet additives, flask content was filtered using Wattman no. 1 filter paper discarding the first few millilitres of filtrate. Ethanol was used for successive dilutions to prepare working tablet concentrations to be assayed adopting our discussed procedure. Table 3 summarizes the calculated tablet nominal contents using the plotted calibration graphs or regression equations.

**Table 3.** Method application to Prostetrol® and Starkoprex® commercial tablets of the studied drugs.

parameter	proposed method		comparison methods [25,36]
	amount taken (ng ml^−1^)	% found^a^	% found^a^
Prostetrol (alfuzosin, 10 mg tab^−1^)	10	101.29	100.53
	20	101.81	98.40
	40	98.02	101.26
	80	100.36	99.60
no. of trials	4		4
mean ± s.d.		100.37 ± 1.68	
Student-*t*		0.40 (2.47)^b^	
*F*		1.85 (9.28)^b^	
Starkoprex (tadalafil, 5 mg tab^−1^)	20	98.27	98.29
	50	101.00	99.14
	80	100.17	101.71
	100	99.71	—
no. trials	4		3
mean ± s.d.		99.79 ± 1.15	99.71 ± 1.78
Student-*t*		0.06 (3.18)^b^	
*F*		2.42 (9.55)^b^	

^a^Each result is an average value of three separate determinations.

^b^The values in brackets refer to tabulated *t* and *F* tests' values.

##### 2.4.2.3. Analysis of AFH and TDF in spiked human plasma and urine

Into a set of 15 ml centrifugation tubes, 1.0 ml aliquots of human plasma or urine were transferred and spiked simultaneously with aliquots of AFH and TDF to have the final studied concentrations as stated (table 1). All tubes were subjected to 1 min vortex mixing and afterward, completed to 5 ml with ethanol that acts as diluting solvent and precipitating agent as well to allow precipitation of plasma proteins. The prepared sets examined a 30 min 3600 r.p.m. centrifugation to separate precipitated plasma efficiently while keeping AFH and TDF analytes in the supernatant layer. Then, 1.0 ml of supernatant was filtered by a 0.2 µm syringe disc filter to remove any suspended particles, transferred to 10.0 ml volumetric flasks, then measurement procedure under §2.4.1 was applied. Table 4 summarizes the final studied concentrations and their corresponding % recoveries. The reported extraction procedure [54] was followed.

**Table 4.** Method application to spiked human plasma and urine samples.

parameter	plasma samples				urine samples			
	amount taken (ng ml^−1^)		% recovery		amount taken (ng ml^−1^)		% recovery	
	AFH	TDF	AFH	TDF	AFH	TDF	AFH	TDF
mixture	10	10	106.93	99.24	10	10	83.69	100.79
	30	30	86.35	106.1	20	30	105.19	97.58
	50	50	106.66	99.13	60	70	97.54	102.85
	70	70	102.66	93.96	80	100	102.12	98.72
	90	100	98.16	102.58				
mean			100.15	100.20			97.14	99.99
± s.d.^a^			8.50	4.50			9.50	2.33
slope			−0.26	−0.07			3.88	1.27
intercept			−0.25	−1.25			−0.53	166.89
*S*_y/x_			0.90	0.19			5.23	4.34
*S*_a_			0.78	0.09			3.42	2.96
*S*_b_			0.01	0.002			0.09	0.06

^a^Average of three separate estimations.

## 3. Results and discussion

Spectrofluorometry as a technique has the merits of high sensitivity and modest selectivity that could be used for the detection of different compounds in biological samples. When it came to mixture analysis, it was noticed that some assayed mixtures suffer from spectral imbrication; hence, different separation techniques should be tried to achieve well-resolved peaks for the assayed drugs [55,56].

As reported, either recording emission spectra or the synchronous technique could help resolve overlapped analytes' peaks [55,56]. The studied AFH and TDF mixture suffered from spectral imbrication, then different separation trials were examined.

**Figure 2.**
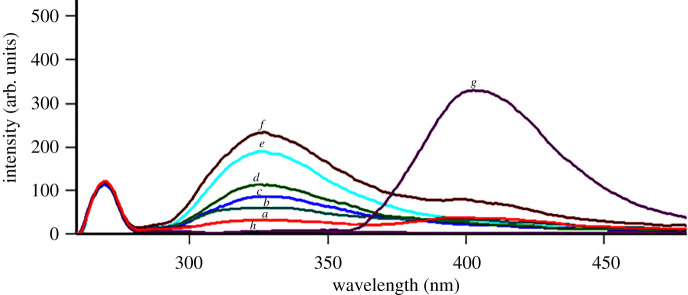
Emission spectra of (*a–f*). TDF; 10, 20, 40, 50, 80 and 100 ng ml^−1^, (*g*): AFH; 6 ng ml^−1^ and (*h*); blank ethanol. All measurements were performed at an excitation wavelength of 250 nm.

First, overlaid emission spectra of AFH and TDF were recorded following a stepwise excitation using a range of 210–320 nm wavelengths. Upon applying 250 nm excitation wavelength, resolution of TDF emission spectra was completely observed at 330 nm without overlap with AFH peaks as AFH showed zero-emission at this wavelength. Unfortunately, the same excitation wavelength (250 nm) showed an emission peak of AFH at 400 nm that suffered from an overlap with the TDF emission peak hindering the calibration of AFH at this scan as shown in figure 2.

**Figure 3.**
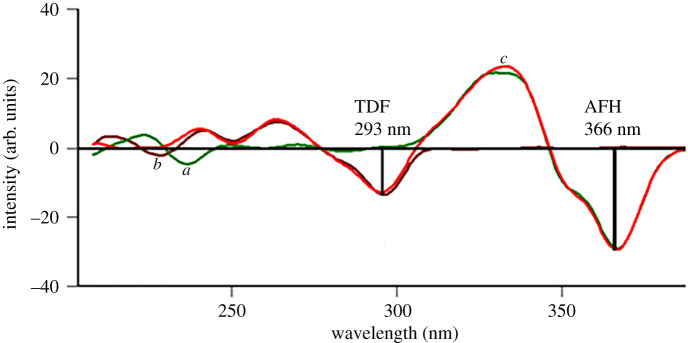
Superimposed first derivative integrals of synchronous fluorescence spectra Δ*λ* of 60 of: (*a*) AFH (60 ng ml^−1^), (*b*) TDF (60 ng ml^−1^) and (*c*) a synthetic mixture of both drugs at the same concentration (60 ng ml^−1^).

Second, another scanning mode was examined and the first-derivative synchronous spectrofluorometry was investigated. Where a stepwise study of synchronous peaks of the studied drugs was done to resolve AFH peaks from TDF ones. Different Δ*λ* values ranging from 20 to 160 nm have been tried and Δ*λ* of 60 nm was the choice. This specific Δ*λ* was chosen for AFH calibration due to high sensitivity besides more than tenfold calibration range (5–90 ng ml^−1^) and a tenfold range of TDF (10–100 ng ml^−1^). Upon applying zero crossing first-order to synchronous peaks obtained at Δ*λ* of 60 nm, well-resolved peaks were detected. TDF showed a zero-crossing wavelength for AFH: 293 nm, at which TDF measurements were recorded, meanwhile AFH had a zero-crossing wavelength for TDF of 366 nm at which AFH peaks were calibrated as in figure 3. In order to evaluate the applicability of the selected zero-crossing point, a synthetic mixture of both drugs was prepared and two single preparations of AFH and TDF, all of the same concentration of 60 ng ml^−1^, they were then subjected to the first derivative synchronous spectrofluorometric scan and superimposed as shown in figure 3. The selected 293 nm showed the same intensity of TDF in its single and synthetic measurements, confirming the optimum selection of the zero-crossing wavelength for further TDF application. For AFH, two wavelengths could be chosen, 345 and 366 nm. Calibrations of AFH were performed at 366 nm because it showed the same intensity as AFH in its single and synthetic mixture preparations, as depicted in figure 3. It can be stated that Δ*λ* of 60 was utilized for simultaneous quantitation of both analytes in their mixtures.

The presented study offers an alternative method that allows resolving overlapped spectra without prior sample treatment or separation. The investigated method showed a satisfying sensitivity for detection of TDF and AFH in human plasma samples spiked with them. It was reported by pharmacokinetic studies that AFH showed concentrations of 2–12 ng ml^−1^ in plasma and 1–5 ng ml^−1^ in urine [57], while TDF showed plasma concentrations of 50–320 ng ml^−1^ and urine concentration of 25 ng ml^−1^ [58].

Additionally, ethanol was selected as the optimum diluting solvent since it reinforced green chemistry guidelines which are recently established to minimize occupational and environmental hazards [51]. Ethanol was the efficient precipitating agent that helped remove plasma proteins during method application to spiked human biological samples. It was superior to methanol that was not able to completely precipitate plasma proteins and this has been proven by high values of blank methanol.

A summarized comparison between the proposed method and some reported ones regarding linear range, LOD, LOQ, applied technique, selectivity and application is detailed in table 5. It can be stated that the proposed method overcame complexity of HPLC methods, high detection limits of ultraviolet spectrophotometry. Moreover, samples were analysed instantaneously by the proposed method with no derivatization step. Ethanol (which is an environmentally compatible solvent) was used by the proposed technique apart from acetonitrile that has environmental hazards and was used by different reported methods.

**Table 5.** Comparison between the proposed method and a side of previous reports. NS: not stated. ACN: acetonitrile. UV: ultraviolet.

parameter	proposed method	reported methods					
AFH							
linear range (ng ml^−1^)	5–90	[15] 0.7850 ng ml^−1^	[16] 250–11 000	[20] 12 500–62 500	[21] 4000–20 000	[25] 50–750	[26] 10–400
LOD (ng ml^−1^)	0.72	0.78	50	682	NS	1.60	1.59
LOQ (ng ml^−1^)	2.18	0.039	150	12 500	NS	4.86	11.76
applied technique	1st-order synchronous spectrofluorometry	RP-HPLC fluorometric detection	HPLC-UV	reaction with ninhydin UV at 575 nm	diazotization with nitrous acid UV at 520 nm	native Fluorescence	derivatization with ortho-phthalaldehyde, spectrofluorometry *λ*_ex_ 337.0 nm and *λ*_em_ 430.0 nm
selectivity	method tolerance was discussed	AFH resolved from plasma	AFH resolved from degradation products	NS	NS	selective	reagent reacts with NH_2_
application	tablets, plasma and urine	plasma	tablets	tablets	tablets	tablets	human plasma
main drawback		use ACN	t_R_ = 10.7 min; utilize ACN	tedious derivatization; high LOD	tedious derivatization; high LOD	high LOQ	derivatization costs time
TDF	proposed method	reported methods					
linear range (ng ml^−1^)	10–100	[32] 4000–80 000	[33] 10 000–150 000	[35] 100–12 000	[35] 20–100	[36] 4–40	[37] 10–50
LOD (ng ml^−1^)	0.57	980	NS	30	5.76	1	0.24
LOQ (ng ml^−1^)	1.72	2960	NS	90	17.09	4	0.70
applied technique	1st-order synchronous spectrofluorometry	HPLC- UV at 260 nm	HPLC- UV at 285 nm	coupling with gold nanoparticles UV at 660 nm	coupling with gold nanoparticles spectrofluorometry *λ*_ex_ 455 nm and *λ*_em_ 489 nm	native fluorescence *λ*_ex_ 280/*λ*_em_ 330 nm	native fluorescence *λ*_ex_ 315/*λ*_em_ 332 nm
selectivity	method tolerance was discussed	separation of TDF from ambrisentan and their degradation products	NS	common tablet excipients did not interfere to a certain drug concentration		no interference from tablet excipients	selective
application	tablets, plasma and urine	tablets	tablets	tablet	tablet and spiked plasma	tablet and spiked plasma	spiked human plasma
main drawback		t_R_ 7.10 min m; ph CH_3_OH and ACN 60%	ACN 50%	stand 5.0 min before measurement		ACN diluting solvents	solvent 0.1 M methanolic H_2_SO_4_

### 3.1. Method optimization

Spectrofluorometry is a sensitive technique where different factors may affect the RFI of AFH and TDF. The carefully optimized affecters included pH, surfactants of different types, and diluting solvent to achieve the best linearity, sensitivity and stability. Univariate optimization was used by carefully studying each affector, keeping the other two affecters constant.

#### 3.1.1. Effect of pH

Using a buffering agent may have an impact on the fluorescence spectra, in other words, it could enhance RFI or plummet it. This depends mainly on the analyte's resonance forms that depend on its chemical structure and pH values [59].

Therefore, pH values ranging from 3.5 to 10 were examined. Neither AFH nor TDF showed a remarkable elevation of RFI in the studied range of pH. It was noticed that upon using lower pH values TDF's RFI decreased, while higher pH values decreased the RFI of AFH. Hence, no buffering system was needed for the presented method and its applications.

#### 3.1.2. Effect of surfactants

**Figure 4.**
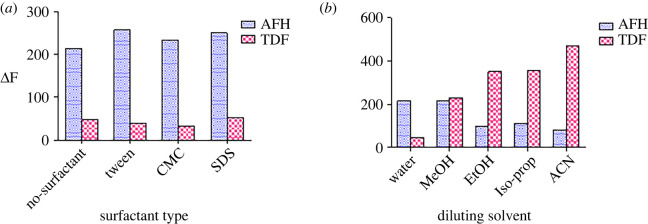
Effect of (*a*): surfactant types, and (*b*): diluting solvents, on relative fluorescence intensity of the studied analytes at Dλ of 60 nm using AFH: 10 ng ml^−1^ and TDF: 100 ng ml^−1^.

The magnitude of the fluorescence emission is greatly impacted by both force and number of collisions taking place in the solution. Collisions are responsible for scattering the fluorescence emission by promoting radiationless decay; then extra energy will be lost as heat. More viscous solutions are supposed to have fewer collisions and minimize radiationless decay [59]. A study of different surfactant types was set using tween 80, sodium dodecyl sulfate (SDS), carboxymethylcellulose (CMC) and 1 gm% of each. Both CMC and tween 80 were excluded due to high blank values while SDS was rejected due to minimizing TDF's RFI, although it showed enhancement of AFH (figure 4*a*).

#### 3.1.3. Choice of diluting solvent

The nature of diluting solvents is different; therefore, they possess different effects on RFI of the studied analytes. For instance, when solvent forms hydrogen bonds, the *λ*_max_ of both emission and excitation spectra may be altered, then changing the energy levels of electrons in π* orbitals and non-bonding electrons [59]. That is the reason why different solvents were investigated in our study. The investigated solvents involved distilled H_2_O, acetonitrile, isopropanol, ethanol and methanol. As illustrated in figure 4*b*, ethanol was the diluting solvent of choice due to its sevenfold enhancement of TDF's RFI compared with distilled water and keeping satisfying intensity for AFH. Although acetonitrile showed more increase in TDF's RFI, it was avoided due to its occupational and ecological hazards in comparison with ethanol, which is considered a green solvent.

#### 3.1.4. Impact of time and temperature

The stability of RFI of studied materials could be altered by changes in temperature and time. The fluorescence intensity of sample solutions is significantly affected by temperature, so it is important to make sure that all the measurements are done at the same temperature. Higher temperatures are thought to speed the movement of the molecules up, leading to more collisions and a decline of the fluorescent intensity. On the other hand, decreasing the temperature of the samples may increase the signal-to-noise ratio [59]. Practically, upon studying RFI of AFH and TDF, the room temperature was the condition of choice minimizing the potential complexity of method as higher temperatures raised potential of ethanol evaporation that may alter sample concentration, while lower temperatures could not be applied as the used device lacked thermal isolation. About time, measurements were recorded over 24 h and noticed to be instant and showed 24-hour stability at room temperature.

### 3.2. Greenness assessment by green analytical procedure index, analytical Eco-scale, and analytical GREEnness metric approach

Analytical Eco-scale is considered a semi-quantitative greenness tool for both laboratory practice and educational purposes because it does not provide comprehensive data concerning the evaluated protocol [50,51]. The idea of the Eco-scale is to calculate penalty points for reagents and instruments using the hazard pictograms, then the total Eco-score is calculated by subtracting total penalty points out of 100 as shown in table 6. The proposed method showed a total Eco-score of 96. The method is excellent green according to Eco-scale as values above 75 are considered excellent green.

**Table 6.** Assessment of proposed spectrofluorometric method greenness using penalty points of Analytical Eco-scale.

reagents	total penalty points [50]
ethanol (10 ml/sample)	1 × 1 = 1
instrument	
energy	0 (≤0.1 kWh per sample)
occupational hazard	0 (no vapours)
waste	3 (no treatment)
total penalty points	4
analytical Eco-scale total score	96

**Figure 5.**
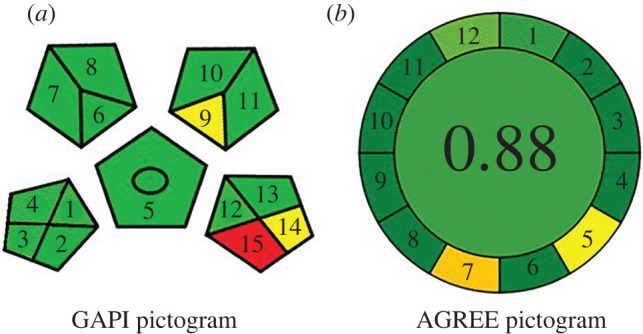
(*a*) GAPI for greenness assessment and (*b*) obtained AGREE graph for the proposed method.

Green analytical procedure index (GAPI) is a diagrammatic tool that aims to describe the greenness of the entire procedure. Herein, five pentagrams are divided into 15 sections, each pentagram corresponds to sample preparation, reagents and compounds used, sample collection, instrumentation and general method type. Each pentagram is subdivided into different sections that represent sub-steps under the pentagram title [51] as shown in figure 5*a*. Additionally, only small amounts of ethanol were needed per sample throughout method application with limited amounts of waste. The proposed method could be used for quantification and qualification procedures.

Analytical GREEnness metric approach (AGREE method) took assessment criteria from the 12 green analytical chemistry principles and converted them into a 0–1 scale. The calculated final score is based on the 12 principles using a given software. The result is a pictogram indicating the final score from 0 to 1, the method is greener when the calculated values are closer to 1 [52] figure 5*b*.

### 3.3. Validation

#### 3.3.1. Linearity and range

By plotting ΔF values against the corresponding concentrations in ng ml^−1^, calibration curves were obtained. Linearity ranges were observed over the concentration ranges of 5–90 and 10–100 ng ml^−1^ for AFH and TDF, respectively (table 1). By analysis of the data obtained, AFH and TDF had the following regression formula:

AFH: ΔF=−19.07+9.89C, TDF: ΔF=−1.29+2.20C.

C: drug concentration (ng. ml^−1^), ΔF: relative fluorescence intensity.

In addition, linearity of calibration graphs was also confirmed by the results of statistical analysis of the obtained data [60] that proved linearity of the calibration curves by values of (*r*) that signifies correlation coefficient. As AFH and TDF showed correlation coefficient values of 0.9998 and 0.9999, respectively (table 1).

#### 3.3.2. Detection and quantitation limits

Both values were calculated by data obtained in calibration graphs following ICH guidelines [53] using the reported equations: LOD = 3.3 *S*_a_/slope, and LOQ = 10 *S*_a_/slope, where LOD is the limit of detection, LOQ is the limit of quantitation and *S*_a_ is the standard deviation of intercept. Table 1 summarized values of LOD and LOQ for AFH and TDF.

#### 3.3.3. Accuracy and precision

The accuracy of the proposed procedure could be expressed by accepted values of the Student *t*-test and variance ratio *F*-test when compared with comparison procedures. Obtained data and analysis values are expressed in table 2.

AFH comparison method was based on direct spectrofluorometric emission measurement at 390 nm after excitation at 325 nm in deionized water with reported linearity of 50–750 ng ml^−1^ [25]. It is noticed that the developed method is more sensitive than the reported one for AFH. For TDF, its comparison method had the same technique as emission spectra were measured at 330 nm upon excitation at 280 nm in acetonitrile with reported linearity of 4–40 ng ml^−1^ [36]. The proposed method used ethanol instead of acetonitrile in TDF's procedure, so the proposed procedure is more eco-friendly in this manner.

Table 7 recaps the practically obtained inter-day and intra-day precision results for the proposed method. Low values of % RSD and % error indicated reasonable intra- and inter-day precision.

**Table 7.** Precision of the proposed method for the determination of AFH and TDF in their raw materials.

sample concentration (ng. ml^−1^)	% found^a^ (intra-day precision)	% found^a^ (inter-day precision)	sample concentration (ng. ml^−1^)	% found^a^ (intra-day precision)	% found^a^ (inter-day precision)
	AFH			TDF	
40	101.35	99.08	20	101.01	98.5
	98.57	100.34		100.78	100.78
	98.06	101.86		100.78	101.69
X′ ± s.d.	99.31 ± 1.77	100.41 ± 1.39	X′ ± s.d	100.85 ± 0.13	100.30 ± 1.64
% RSD	1.78	1.39	% RSD	0.13	1.64
% error	1.03	0.80	% error	0.08	0.94
60	99.22	100.07	50	100.38	99.47
	99.9	101.41		101.74	98.56
	100.07	100.91		99.01	101.29
X′ ± s.d.	99.73 ± 0.45	100.79 ± 0.68	X′ ± s.d	100.36 ± 1.37	99.76 ± 1.39
% RSD	0.45	0.67	% RSD	1.36	1.39
% error	0.23	0.39	% error	0.79	0.80
80	101.06	100.59	80	99.14	98
	100.33	101.34		100.85	100.28
	100.84	101.47		101.42	101.99
X′ ± s.d.	100.74 ± 0.37	101.13 ± 0.48	X′ ± s.d	100.46 ± 1.19	100.06 ± 2.00
% RSD	0.37	0.47	% RSD	1.18	2.00
% error	0.21	0.27	% error	0.68	1.16

^a^Average of three individual assays.

#### 3.3.4. Selectivity

The selectivity of the proposed method was investigated by application of the proposed method to certain drugs that may be co-administered in accompanied diseases such as hypertension, type II diabetes mellitus and obesity [61,62]. The tested drugs are hydrochlorothiazide, spironolactone, indapamide, metformin, orlistat, captopril and nicardipine, and the results indicated the high selectivity of the proposed method by lack of RFI of these drugs under the optimized conditions of study.

#### 3.3.5. Stock and working solutions stability

Over a month, the % found of 50.0 ng ml^−1^ solutions of AFH and TDF were recorded. The solutions were stable over three successive weeks when refrigerated. As after three weeks, the recorded % found were noticed to be lower than 98.00%.

## 4. Conclusion

A fast, simple, green and sensitive spectrofluorometric method was evolved for the analysis of AFH and TDF in different matrices. The assay principle was based on first-order synchronous spectrofluorometric scan using Δ*λ* = 60 nm in which AFH peaks were recorded at 366 nm and TDF measurements were recorded at 293 nm after converting synchronous scans into their first derivative without overlap. The method was applied to assay the analytes of interest in pharmaceutical dosages. In addition, AFH and TDF were analysed in human plasma samples with % recoveries of 100.15% and 100.20% in spiked plasma for AFH and TDF, respectively. Moreover, the evolved method was proven to be environmentally green by using Analytical Eco-scale, Green Analytical Procedure Index, and GREEnness Metric Approach tools for greenness evaluation.

## Data Availability

Data are available from the Dryad Digital Repository: https://doi.org/10.5061/dryad.7m0cfxpwq [63].
